# Enhanced light microscopy visualization of virus particles from Zika virus to filamentous ebolaviruses

**DOI:** 10.1371/journal.pone.0179728

**Published:** 2017-06-26

**Authors:** George G. Daaboul, David S. Freedman, Steven M. Scherr, Erik Carter, Alexandru Rosca, David Bernstein, Chad E. Mire, Krystle N. Agans, Thomas Hoenen, Thomas W. Geisbert, M. Selim Ünlü, John H. Connor

**Affiliations:** 1 nanoView Diagnostics Inc., Boston, MA, United States of America; 2 Department of Mechanical Engineering, Boston University, Boston, MA, United States of America; 3 Department of Microbiology, Boston University School of Medicine, Boston, MA, United States of America; 4 Galveston National Laboratory, Galveston, TX, United States of America; 5 Department of Microbiology, Galveston, TX, United States of America; 6 Immunology, University of Texas Medical Branch, Galveston, TX, United States of America; 7 Laboratory of Virology, Division of Intramural Research, National Institute of Allergy and Infectious Diseases, National Institutes of Health, Rocky Mountain Laboratories, Hamilton, MT, United States of America; 8 Institute of Molecular Virology and Cell Biology, Friedrich-Loeffler-Institut, Greifswald–Isle of Riems, Germany; 9 Department of Electrical Engineering, Boston University, Boston, MA, United States of America; 10 Department of Biomedical Engineering, Boston University, Boston, MA, United States of America; 11 Physics Department, Boston University, Boston, MA, United States of America; Division of Clinical Research, UNITED STATES

## Abstract

Light microscopy is a powerful tool in the detection and analysis of parasites, fungi, and prokaryotes, but has been challenging to use for the detection of individual virus particles. Unlabeled virus particles are too small to be visualized using standard visible light microscopy. Characterization of virus particles is typically performed using higher resolution approaches such as electron microscopy or atomic force microscopy. These approaches require purification of virions away from their normal millieu, requiring significant levels of expertise, and can only enumerate small numbers of particles per field of view. Here, we utilize a visible light imaging approach called Single Particle Interferometric Reflectance Imaging Sensor (SP-IRIS) that allows automated counting and sizing of thousands of individual virions. Virions are captured directly from complex solutions onto a silicon chip and then detected using a reflectance interference imaging modality. We show that the use of different imaging wavelengths allows the visualization of a multitude of virus particles. Using Violet/UV illumination, the SP-IRIS technique is able to detect individual flavivirus particles (~40 nm), while green light illumination is capable of identifying and discriminating between vesicular stomatitis virus and vaccinia virus (~360 nm). Strikingly, the technology allows the clear identification of filamentous infectious ebolavirus particles and virus-like particles. The ability to differentiate and quantify unlabeled virus particles extends the usefulness of traditional light microscopy and can be embodied in a straightforward benchtop approach allowing widespread applications ranging from rapid detection in biological fluids to analysis of virus-like particles for vaccine development and production.

## Introduction

Viruses are a diverse group of pathogens that have taken widely different life-cycle and genome storage approaches. Perhaps unsurprisingly, assembled viral particles that make up the infectious unit are highly diverse in shape and size. Virion sizes can range from 400 nm in diameter for DNA viruses such as mimivirus and poxviruses to small virions of ~25nm for polio virus. Morphology can also vary widely, from filamentous ebolavirus and marburgvirus virions to pleomorphic viruses such as measles to highly regular “brick” shaped poxvirions.

Virus morphology can be unique enough to enable diagnosis [1], and can be an important factor in individual virion infectivity. One example of this has come from careful analysis of filoviruses, where detailed studies have shown that filamentous marburgvirus and ebolavirus particles are more infectious than circular particles that are also produced by infected cells [2,3]. A second example of morphology impacting virus function has come from studies of influenza A virus. Though influenza A virions are often described as spherical, it is becoming increasingly noted that clinical samples have a large number of filamentous virions and that the spherical particle form of the influenza A virion is selected for passing in cell culture passaging [4].

The evolution in understanding virus morphology and its impact on influenza and filovirus infectivity is an important example that highlights the limitations of current approaches for “seeing” virions. Various approaches currently exist to analyze virus particles, including fluorescent flow cytometry, dark field microscopy of purified particles, electron microscopy (EM), and atomic force microscopy (AFM) [1,5–9]. While all of these approaches have various strengths, they can be more challenging to implement and interpret than light microscopy and require significant sample preparation prior to use that can have an impact on what is visualized [10]. It is recognized that the preparation of viruses by ultracentrifugation and other methods to facilitate EM imaging can destroy filamentous virions [10,11], leaving spherical particles or broken filaments [3] as the visible particles. These limitations highlight the need for different approaches that enable the detection and characterization of individual virion particles.

One viable approach that can be used to detect and analyze virions is a recently described nanoparticle detection system that uses reflectance interference based light microscopy (SP-IRIS) to count and size nanoparticles (e.g. virions) captured from various fluid samples ranging from cell-culture media to blood [12]. SP-IRIS has also been shown to be able to quantify virus concentrations from 10^2^–10^6^ PFU/mL directly from cell culture media and when spiked into serum [13]. Furthermore, for diagnostic applications it was recently shown the SP-IRIS assay can be run passively in an enclosed lateral flow assay in under 30 minutes with sensitivity better than ELISA and rapid antigen tests [14].

This approach has been used to directly detect unlabeled influenza A virus and VSV particles (~100 nm particles) [13,15–17], but the ability of the technology to detect larger (~400 nm), smaller (~50 nm) and filamentous particles had not been investigated. Here we describe the results of experiments that tested the utility of SP-IRIS for the detection of different size virions. We show that the use of two illumination sources allows the detection of virions ranging from Zika virus (40 nm) to poxvirus virus particles (~400 nm). We also show that this approach allows the characterization of virion populations of diverse shapes, and can distinguish filamentous particles from non-filamentous particles in a quantitative manner. This approach for virus detection can sit on a benchtop and is easily used, offering a new approach for analyzing unlabeled viruses in a variety of different conditions.

## Materials and methods

### SP-IRIS Imaging and particle analysis

SP-IRIS images were taken with the NVDX10 automated reader by nanoView Diagnostics. The reader illuminates the sensor chip with either 420 nm or 535 nm wavelength light from an LED light source. The imaging system uses a 40X 0.75 N.A objective and a CMOS camera to record the images to the computer. SP-IRIS images are analyzed by custom software written by nanoView Diagnostics. For diffraction limited particles the software automatically detects the sub-diffraction limited point features in the image and calculates the contrast based on the local particle-free background. The calculated contrast is then converted to a diameter using a 3^rd^ order polynomial function. For filamentous particle detection the software searched for objects that are diffraction limited in one dimension and starting with the center of the object, iteratively fit one-pixel at a time, the best shape of the object. The best shape was determined by convolving a point-spread function with the pixel shape of the object and placing a new pixel if the maximum 2d correlation-coefficient increased. This step was repeated in the reverse-direction of the center of the object. The final pixel length of the shape was found by closing the object, shrinking to the minimum diameter, and then removing any spurious pixels. The calculated length was then found by multiplying the pixel length by the size of the magnified image on the pixel, i.e. 147 nm/pixel.

Shot-noise, resulting from the collection of discrete photons on the sensor, is the dominant source of noise in the system and therefore the limiting factor when detecting dim objects against the background level of a brightfield microscopy image. The theoretical limit of shot-noise correlates with the square root of the number of photons being collected by the camera sensor. By averaging multiple images, the effective number of photons collected by the sensor is increased, reducing shot-noise [18]. For detection of nanoparticles above 70 nm we average 32 frames. For detection of small nanoparticle the shot noise of the system was reduced to below 0.1% contrast through averaging at least 128 images of SP-IRIS data. For detection ZIKV 512 images were averaged.

### Sensor fabrication and printing

The SP-IRIS chip consists of a silicon substrate with a thin silicon dioxide top layer. The glass top layer is compatible with standard glass chemistries. For the SP-IRIS virus capture assay, the sensors are functionalized with a copolymer with reactive NHS groups to bind antibody probes [19]. The antibody microarrays are robotically printed using the S3 Flexarrayer (Scienion AG). The S3 is a non-contact spotter that allows dispensing of user defined volumes of reagent on the surface as low as 120 picoLiters. Each antibody probe is spotted at high concentration (> 2.0 milligrams/mL) in PBS. After 24 hour incubation in a humid chamber any unbound antibodies was washed with PBST (1X PBS with .1% Tween-20) followed by rinse with Millipore water and dried. The printed chips are quality checked using a label-free method to measure the probe density and morphology of all conditions on each printed sensor before running the assay [20].

For virus capture antibodies against virus surface glycoproteins of ZIKV, VSV, VACV, and EBOV were printed on the sensor’s surface. An anti-EBOV glycoprotein antibody (13F6) was provided by Larry Seitlin at Mapp Biopharmaceutical, San Diego. A mouse monoclonal antibody (8G5) [21] was used for VSV capture. A mouse monoclonal antibody (HB112, ATCC) was used for ZIKV capture. A mouse monoclonal antibody (DD37 AB-VACC-MAB1) from BEI resources was used for VACV capture.

### Virus preparation and incubation

Stocks of Vesicular Stomatitis Virus (VSV)–were prepared by infecting Vero cells at an MOI of 0.1, incubating at 37°C, and harvesting virus-containing media 3 days post-infection. Crude media was spun down for 10 minutes at 4750 rcf to pellet cellular debris, aliquoted, and stored at -80°C for future use. The titer of VSV was determined by plaque assay using Vero cells. A recombinant VSV EBOV was produced with the glycoprotein genes from Ebola virus (Zaire) in place of the native VSV G gene as previously described [22].

Vaccinia virus stocks were generated by sonicating starter virus in a bath sonicator for 20 seconds and infecting HeLa cells at 90–95% confluency (grown in DMEM with 1% L-glutamine and 10% FCS) at an MOI of 0.1. After three days, cells were detached from the tissue culture plate surface with a cell scraper and transferred, in the culture media into a 50 mL conical tube and freeze-thawed three times to lyse the cell. The freeze-thawed media was then spun down for 10 minutes at 4750 rcf to pellet the cellular debris and the supernatant was aliquoted and stored at -80°C. The titer was determined by plaque assay using HeLa cells.

Stocks of the MR766 strain of Zika virus were prepared by infecting 50% confluent Vero cells with a 1:60,000 dilution of inoculum and incubating at 37°C for 5 days. Culture media was then spun down for 10 minutes at 4750 rcf to pellet the cellular debris and the supernatant was aliquoted and stored at -80°C. The titer was determined by a TCID50 assay using Vero cells.

Ebola virus-like particles (VLPs) were produced using a standard PEI transfection protocol. 293T cells were grown to confluency in a 15 cm tissue culture dish and transfected with pCAAGS-driven plasmids encoding Ebola GP, VP40, and NP [23]. Cells were incubated at 37°C for 2 days, then the culture media was harvested and spun down to remove cell debris. This crude culture media containing VLPs was then purified by ultracentrifugation at 25,000 rpm for 1 hour over a solution of 20% sucrose in 10mM Tris. VLP pellet was then resuspended in PBS and stored at -80°C for future use.

Monocistronic and multicistronic miniginome containing Ebola VLPs were produced in 293T cells as previously described [23]. VLP-containing supernatant was concentrated by ultracentrifugation through a 20% sucrose cushion in a SW28 rotor at 83,000 x g and 4°C. Pellets were resuspended and stored at 4°C. VLPs were tested within 1 week of isolation.

Infectious filovirus work utilized Zaire ebolavirus (EBOV) Mayinga H.sapiens-tc/COD/1976/Yambuku-Mayinga (passage 3; Vero 76 (ATCC, CRL-1587) and Zaire ebolavirus Kikwit isolate from CDC (CDC number 807223) (passage3; Vero 76 (ATCC, CRL-1587)).

### Virus capture on SP-IRIS sensor

Chips were incubated with either purified virus in PBS or from crude virus in cell culture media. The virus stock was diluted to approximately 10^6^ PFU/ml (or 10^6^ TCID50/mL for Zika virus) in PBS and incubated on the sensor chip for 2 hours. After virus incubation, the chips were washed in 1X PBST for three minutes, 1X PBS three times for three minutes each, fixed with 2% glutaraldehyde in 1X PBS, rinsed in Millipore purified water and dried. For Ebola virus experiments chips were fixed for 48 hours in 4% formaldehyde then rinsed with Millipore purified water and dried. The chips were then imaged using the NVDX10 reader. In-liquid real-time detection of Ebola viruses was done through flow of cell culture media containing Ebola virus at a flow rate between 2–4 μL/min. The NVDX10 in-liquid reader captures images of the capture spot every 3 minutes for 20 minutes.

### Scanning electron microscopy

The sensor chips were fixed with 2% gluteraldehyde in PBS for 15 minutes, then rinsed in Millipore purified water and dried. The fixed sensor chips were imaged using a Zeiss Supra VP55 at 1.5 kV and 6 mm working distance. The virus particles were visualized using the side detector.

## Results and discussion

**Fig 1:**
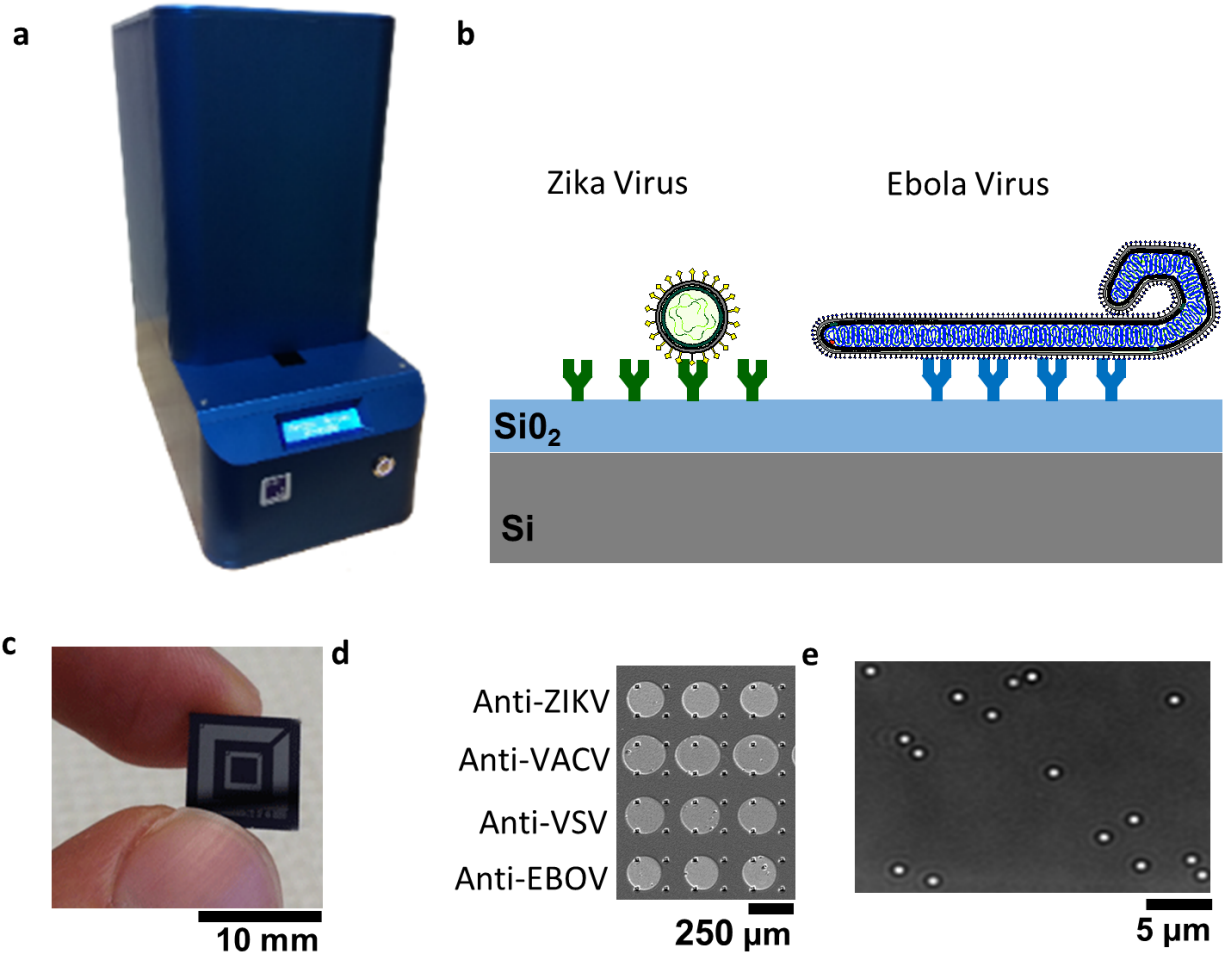
SP-IRIS platform. a) Reader instrument b) cross-selection illustration of chip and antibody assay c) picture of the SP-IRIS chip d) micrograph of label-free quality control assay array image e) example SP-IRIS image illustrating that each bright dot is a single captured nanoparticle.

The detection platform, named Single-Particle Reflectance Imaging Sensor (SP-IRIS) consists of a light-microscopy based reader (Fig 1A) and a sensor chip (Fig 1B and 1C). The sensor chip can be arrayed with different capture probes (Fig 1D) that allow the multiplexed interrogation of a single sample. Captured virus particles can be individually detected up to a density of about 200,000 particles per square millimeter, allowing the robust characterization of each captured viral particle. Briefly, for viruses under the resolution limit of the microscope, which is approximately 435 nm and 340 nm for illumination wavelengths of 535 nm and 420 nm respectively, the peak intensity from each particle in the SP-IRIS image (Fig 1E) is normalized by the local background and used to calculate the particle diameter assuming a spherical object. In this article we present SP-IRIS being used to detect smaller biological particles, i.e. viral particles as small as 40 nm, through improvements increasing the signal-to-noise ratio (SNR). Additionally, we present direct detection of filoviruses, to allow high-throughput characterization of filamentous viruses like the Ebola virus.

Early experiments showed that using a single wavelength illumination approach (535 nm) for virus identification would not allow imaging of small virions such as the flaviviruses (approximately 40 nm in diameter). We therefore added a second, shorter wavelength illumination source at 420 nm (Violet/UV) to improve the amount of scattered light being collected from very small nanoparticles.

**Fig 2:**
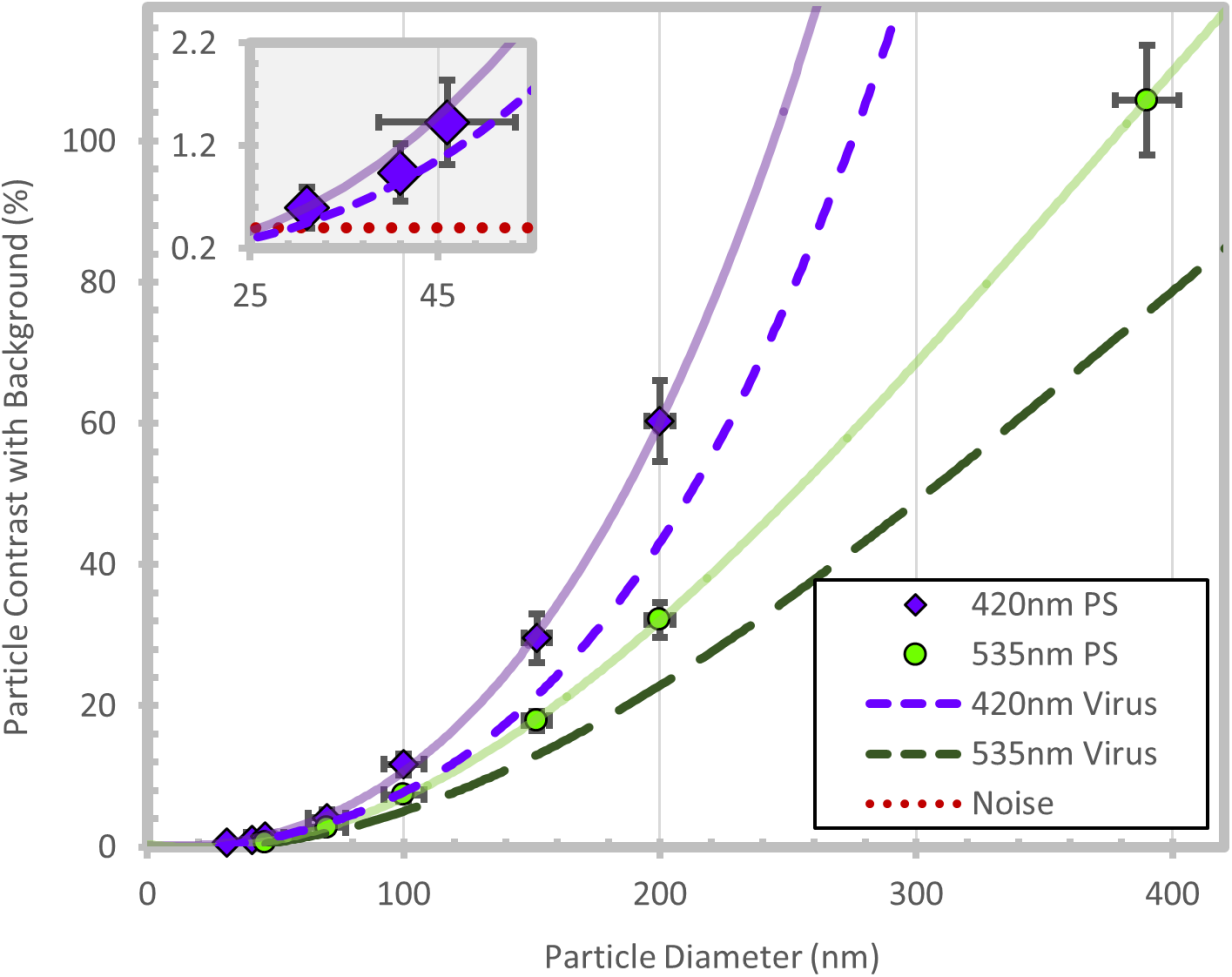
SP-IRIS nanoparticle response. Contrast of detected nanoparticle standards for 420 nm and 535 nm wavelengths are plotted. A cubic polynomial fit of the data is used to size other nanoparticles. The dashed line is used for sizing viral particles with refractive index adjusted to n = 1.42. The dotted redline indicates the noise floor of the system measured at 0.5%. The plotted error bars are +/- one standard deviation.

To validate the nanoparticle size detection sensitivity of the SP-IRIS as shown in Fig 2, polystyrene (PS) particles ranging from 30 nm to 400 nm were imaged and validated using SEM (S1 Fig). The solid purple and green lines are cubic polynomial fits to the measured contrast versus particle diameter for 420 nm and 535 nm wavelength LED illumination, respectively.

Using 535 nm (green) light as the illumination source, a large range of particles can be accurately sized, but particles below 50 nm in diameter are difficult to detect and size [17]. For smaller particles, a shorter wavelength of visible light (420 nm) demonstrated the ability to size particles below 50 nm. The noise level of the background is about 0.5%, limiting particle detection to about 30 nm (S2 Fig). For accurate sizing of virus particles, as compared to PS, the sizing curves were scaled based on the refractive index of PS (n = 1.55) to virus (n = 1.42). The value of n = 1.42 is assumed for each virus and is similar to other label-free optical measurements of virus particles [24,25]. The dashed lines in Fig 2 shows the scaled sizing curves, using the reduced refractive index needed for virus sizing, for both 420 nm and 535 nm illumination. These sizing curves are used to covert the intensity of the virus particle response to particle diameter for every detected particle on the sensor surface.

To test the ability of the system to effectively capture, detect, and characterize different virus particles, printed sensor chips with antibodies specific for the surface glycoproteins of the following viruses were used: Zika virus (ZIKV), Vesicular Stomatitis Virus (VSV), Vaccinia virus (VACV), and Ebola virus (EBOV). These viruses represent a virological ruler of sorts, spanning small (40 nm), medium (100 nm), large (400 nm), and filamentous (90 x 1,000 nm) virus particles.

**Fig 3:**
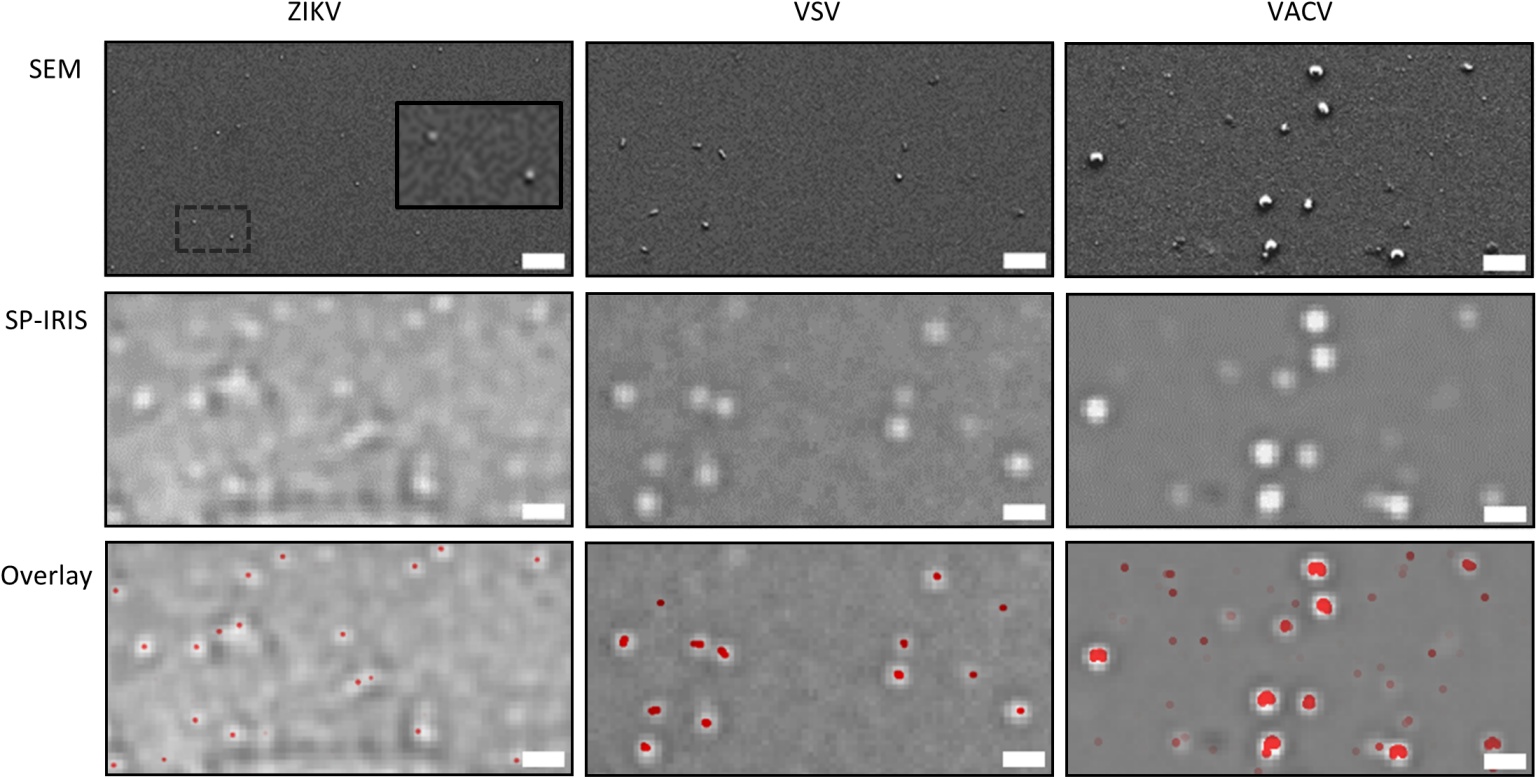
SP-IRIS virus detection validation with SEM. SP-IRIS sensors incubated with ZIKV, VSV, and VACV were imaged with SP-IRIS and SEM. To validate accuracy of virus detection, the same area was imaged with SP-IRIS and SEM. The overlay panel aids in visualizing the correlation between the SP-IRIS and SEM images. Red dots in the overlay image represent particles detected by SEM. Contrasts of the SP-IRIS images were differently enhanced for clarity of presentation.

For each virus, a sensor chip was incubated with virus preparations from Vero cells infected with virus. Viral particles were specifically captured from the virus sample onto the surface of the sensor using the immobilized antibodies specific against the virus surface glycoproteins. Following incubation with the virus sample, the chips were washed, dried, and imaged with both SP-IRIS and scanning electron microscopy (SEM). The SP-IRIS chip is a solid planar substrate that allows the user to image the identical virus capture region on both SP-IRIS and SEM. Fig 3 shows a small region defined by the small field of SEM to validate the capture and detection of viral particles by SP-IRIS.

The top panel of Fig 3 shows SEM images of ZIKV, VSV, and VACV in side scatter mode. The expected size and virus morphology was observed for the different virus samples. However, while the VACV images contained the known “brick” shape of VACV, another subpopulation of smaller incomplete viruses was also seen. The second row of panels shows the SP-IRIS image of the identical area on the chip imaged by SEM. Since the SP-IRIS platform uses visible light microscopy with a resolution of 340-to-435 nm, the viral particles appear as bright dots in the image with the diameter of the viral particle correlating to the brightness of the dot in the image versus the local background (contrast). As expected, as the size of the virus particle from ZIKV to VACV increases, the contrast of the viral particle detected increases. The third row of panels in Fig 3 is an overlay of the SP-IRIS image with the SEM image (colored in red) showing perfect correlation between the two methodologies.

**Fig 4:**
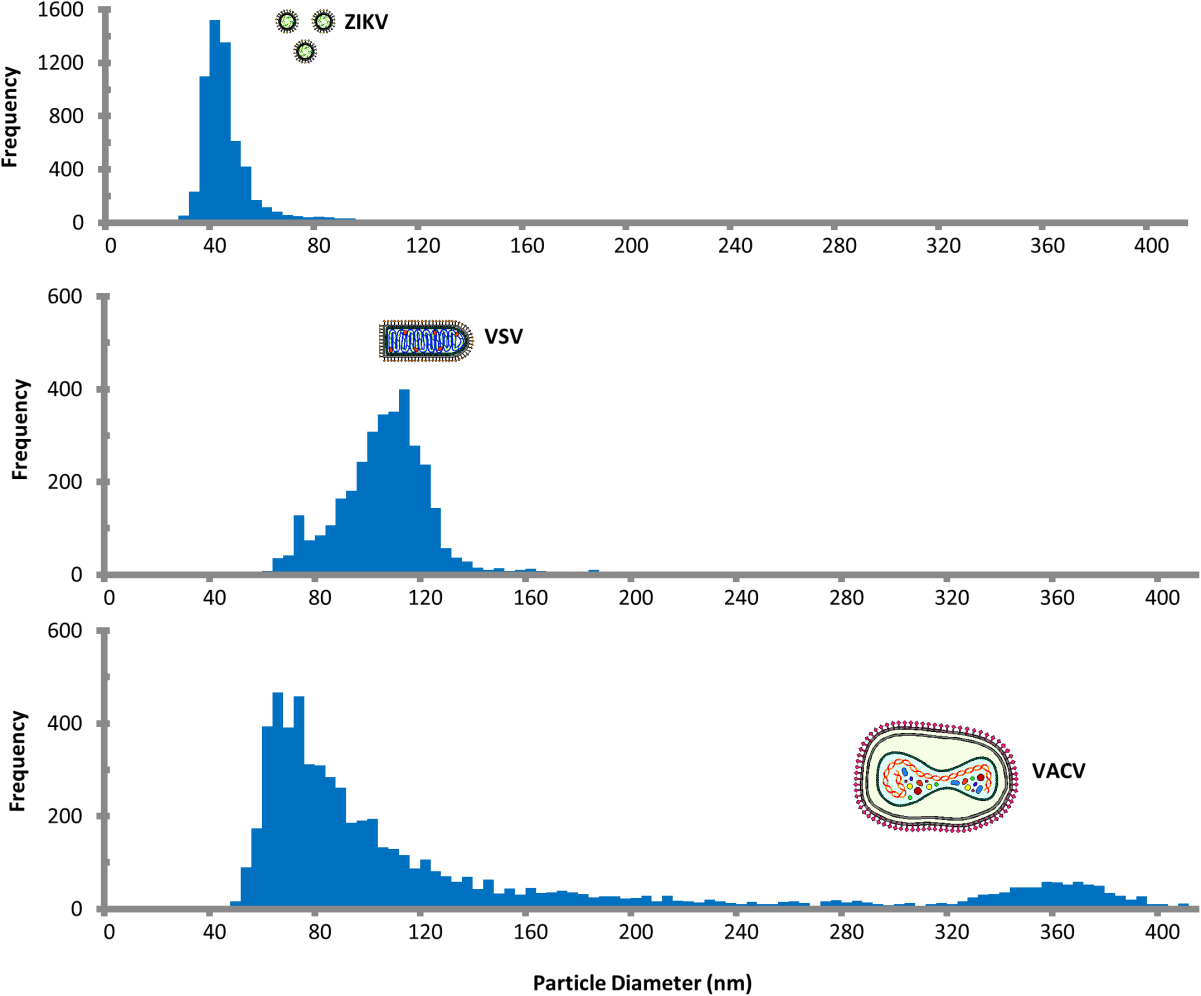
Measured particle distribution of virus preparation measured by SP-IRIS. The contrast of the individual detected particle is converted to particle diameter using the response curve created with polystyrene bead standard and refractive index of the virus.

Fig 4 shows the sizing histograms for ZIKV, VSV, and VACV captured on the sensor surface by antibody spots targeting their surface glycoproteins. Size profiles determined by SP-IRIS matches the sizes reported in the literature [26–28]. Both ZIKV and VSV show a relatively narrow particle size populations centered around 45 nm and 110 nm, respectively, which is expected based on the uniform virus structure observed for these viruses. For VACV, SP-IRIS shows virions that correlate perfectly with the intact virions seen via SEM, and sizing estimates that these virions are 360 nm, which correlates well with the expected size of a poxvirus virion. Importantly, SP-IRIS also detects a second peak of particles at approximately 70 nm, which was also seen in the SEM images. This peak is consistent with the imaging approach capturing partially assembled virion components that are likely present in the crude virus preparation. These data illustrate that SP-IRIS can characterize and quantify significantly different size populations and could likely be used to more quantitatively analyze the virus assembly process.

**Fig 5:**
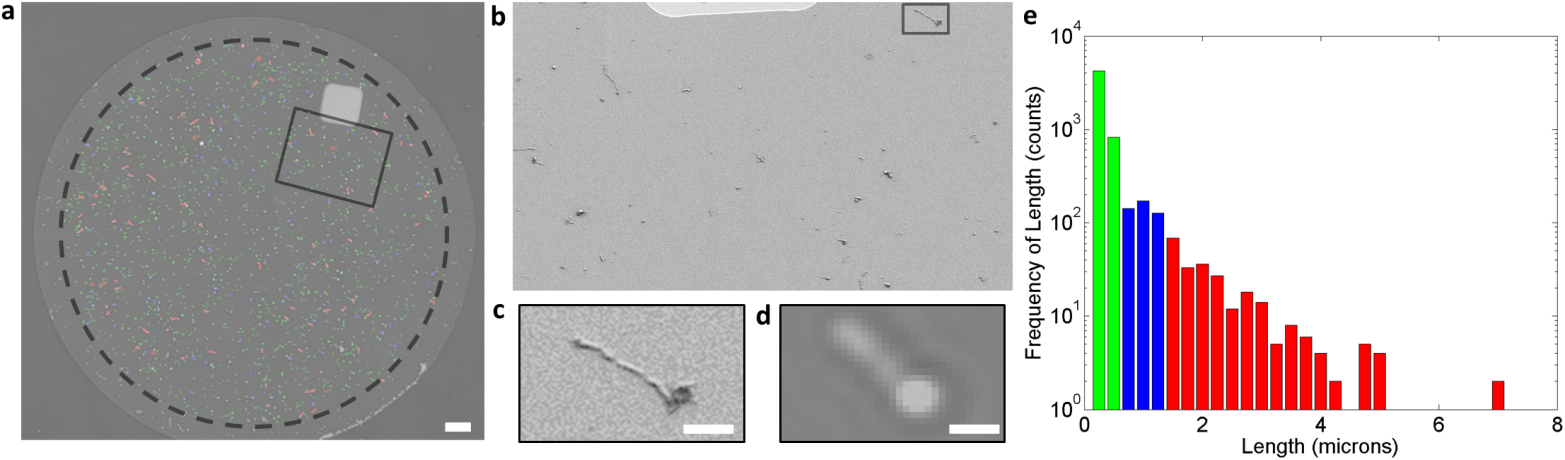
SP-IRIS detection and characterization of filamentous Ebola VLPs. a) SP-IRIS of captured Ebola VLP with identified particles and filamentous color-coded. Scale bar 10 micron. b) SEM of Ebola VLP box, outlined in 5a. c) Single filament outlined in 5b shown for both SEM (c) and SP-IRIS (d). Scale bar 1 micron e) Histogram of Ebola VLP with small (green), medium (blue) and large/long (red) particle frequencies.

Further investigations of the SP-IRIS platform were performed to determine if SP-IRIS technology could be used to visualize filamentous Ebola virus (EBOV) and virus like particles. These particles represent a unique morphology. EBOV is diffraction limited in one dimension (width, ~90 nm) but can be one to several micrometers in length [3]. To validate SP-IRIS capability to detect and characterize filamentous viruses like Ebola, VLPs expressing Ebola GP on its surface were investigated. We first analyzed VLPs generated using VP40, GP_1,2_, and VP24 and a monocistronic minigenome containing a single open reading frame (ORF) [23]. Fig 5A shows the VLPs captured by an anti-EBOV GP antibody. Many individual particles are visible in this image, including filamentous-shaped particles. Fig 5B shows a scanning electron microscopy (SEM) image of a small region of the SP-IRIS spot validating the capture of small nanoparticle and filamentous virions. The SEM image (Fig 5C) compares to SP-IRIS image (Fig 5D), demonstrating agreement between the two techniques. The IRIS image detects the shepherd’s crook shape of the VLP as in increased intensity, so the filament seen in IRIS has a “bulb” on one end. Fig 5E shows a histogram of the detected virions that were automatically color coded by the detection software to indicate VLP length.

Particles are binned into three size regimes as shown by the histogram in Fig 5D: diffraction limited particles are in green, particles smaller than 1.5 μm are in blue, and longer filamentous particles are shown in red. The small particles, labelled in green, are likely the circular or “6”-shaped particles that are commonly formed during virus replication in infected cells, or could represent non-infectious, spherical particles that are found in abundance in EBOV VLP preparations [29]. The particles labelled in blue are filamentous particles with the approximate length of wild-type virions [3]. Filaments labelled in red are long filaments greater than 1.5 μm, which were also seen in EBOV VLP preparations produced without a minigenome (S3 Fig) and in VLP preparations produced by a multicistronic (4cis) minigenome [23] (S4 Fig), and those produced by a multicistronic minigenome lacking a functional VP24 (S5 Fig). All the VLP preparations showed similar distributions between nanoparticles and filamentous virions. A recombinant VSV-EBOV, which contains the EBOV GP but does not form filaments, was used as a negative control for the filamentous particle detection software. These virions, captured using the anti-EBOV GP, showed particles that clustered tightly around a mean of 110 nm diameter same as measured for VSV particles in Fig 4B (S6 Fig).

**Fig 6:**
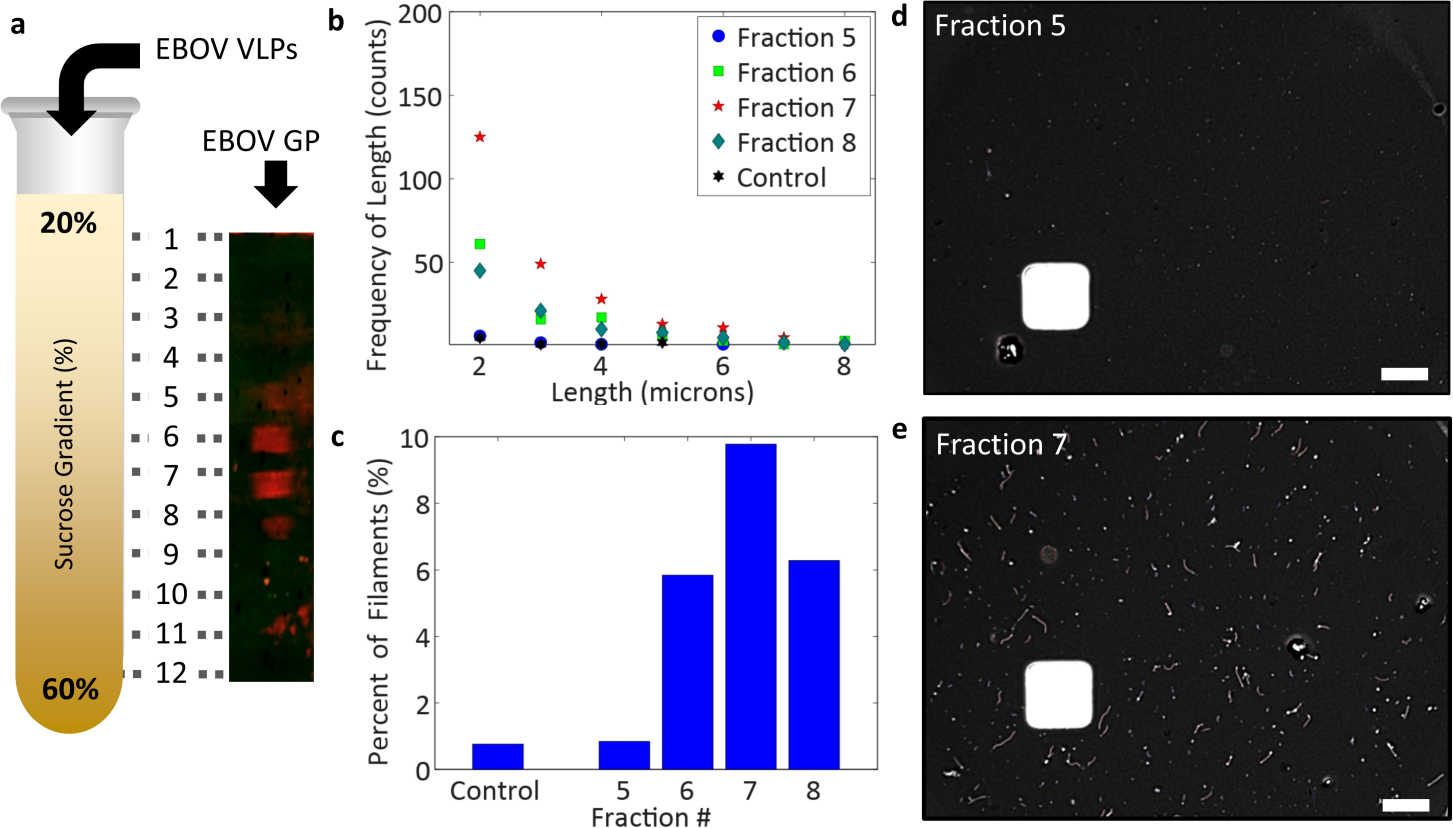
Characterization of sucrose gradient fractionated EBOV VLPs. a) Schematic depiction of Ebola VLPs fractionation on a 20–60% sucrose gradient. Fractions from this gradient were run on an SDS-PAGE gel and VLP was detected with anti-Ebola GP antibody. Fractions 5–8 showed positive signal for Ebola-GP (blot shown at a 90 degree angle to normal viewing) b) Plot of the quantity of Ebola VLP captured on the SP-IRIS sensor by anti-Ebola GP(13F6) vs the size of the detected particles. Fractions shown include 5 (blue circle), 6 (green square), 7 (red star), 8 (green diamond) and a sample with no VLP (black star). c) Graph representing the % of filamentous particles to non-filamentous particles illustrating enrichment of filamentous particles within the gradient. d) individual images illustrating low (top) and high (bottom) filament fractions.

To demonstrate that the SP-IRIS platform can detect differences between virus preparations in terms of concentration and filament size; partially purified EBOV VLPs were further analyzed by centrifugation through a 20–60% sucrose gradient. Fractions which were positive for EBOV GP by western blotting (Fig 6A) were captured on the SP-IRIS sensor using an antibody against EBOV GP and the amounts of filamentous and non-filamentous particles were determined. Fig 6B plots the number of filamentous particles detected for each fraction versus filamentous size. The number of filamentous particles detected correlate with the signal from the western blot, with more larger particles being observed at greater sucrose densities. When the amount of filamentous particles to total particles was determined (Fig 6C), IRIS imaging showed that there was a greater percentage of filamentous VLP in heavier sucrose fractions than in the partially purified VLP preparation (control) or in the lower density fractions. This difference is also readily visible in images (Fig 6D).

**Fig 7:**
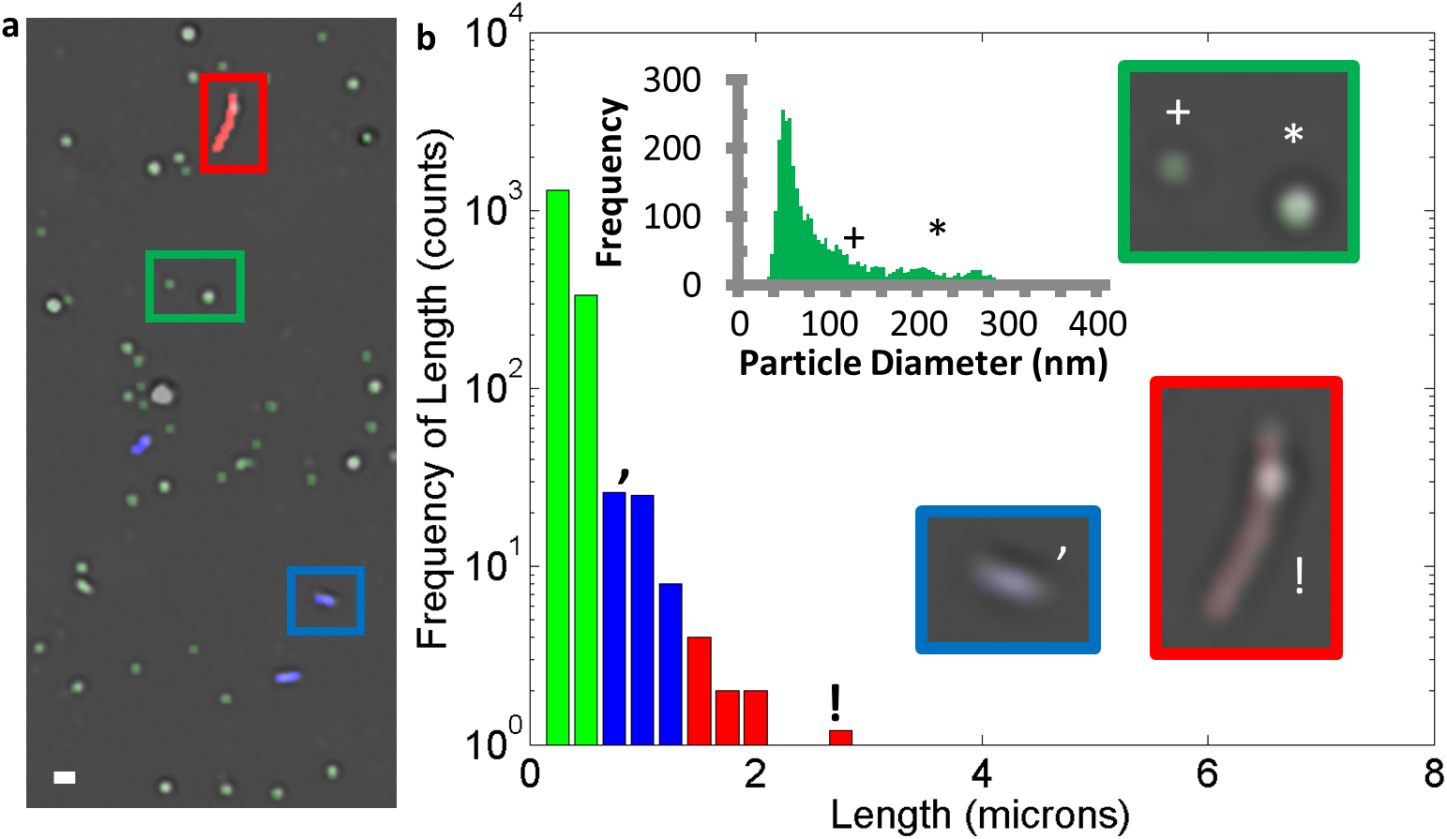
SP-IRIS detection and characterization of Ebola virus. a) SP-IRIS image of captured Ebola virus with identified particles and filaments color-coded. b) Histogram of Ebola VLP with small (green), medium (blue) and large/long (red) particle frequencies. Inset in 5b highlights small, medium and large filaments with size distribution of the diffraction limited small particles. Scale bar is 1 micron.

After verification that the sensor was capturing filamentous VLPs and the software could automatically detect and classify their length, the ability to carry out similar analysis on EBOV (Kikwit isolate) was tested using crude virus preparations. EBOV from Vero culture media diluted with PBS to 10^6^ PFU/mL was incubated for two hours over the SP-IRIS sensor that was functionalized with anti-EBOV GP. Fig 7A shows a selection of the SP-IRIS image that captured EBOV on the anti-EBOV GP antibody spot (S7 Fig) with detected filaments and nanoparticles that are color coded based on size/length. Fig 7B shows the distribution of particle types captured by the anti-EBOV GP spot and examples of representative particles. EBOV samples showed filamentous particles in the 1–2 micron range, but did not show a significant number of longer particles as were observed in VLPs. These results are in keeping with EM experiments that have identified single genome (1 μm) and double-genome containing particles [3].

**Fig 8:**
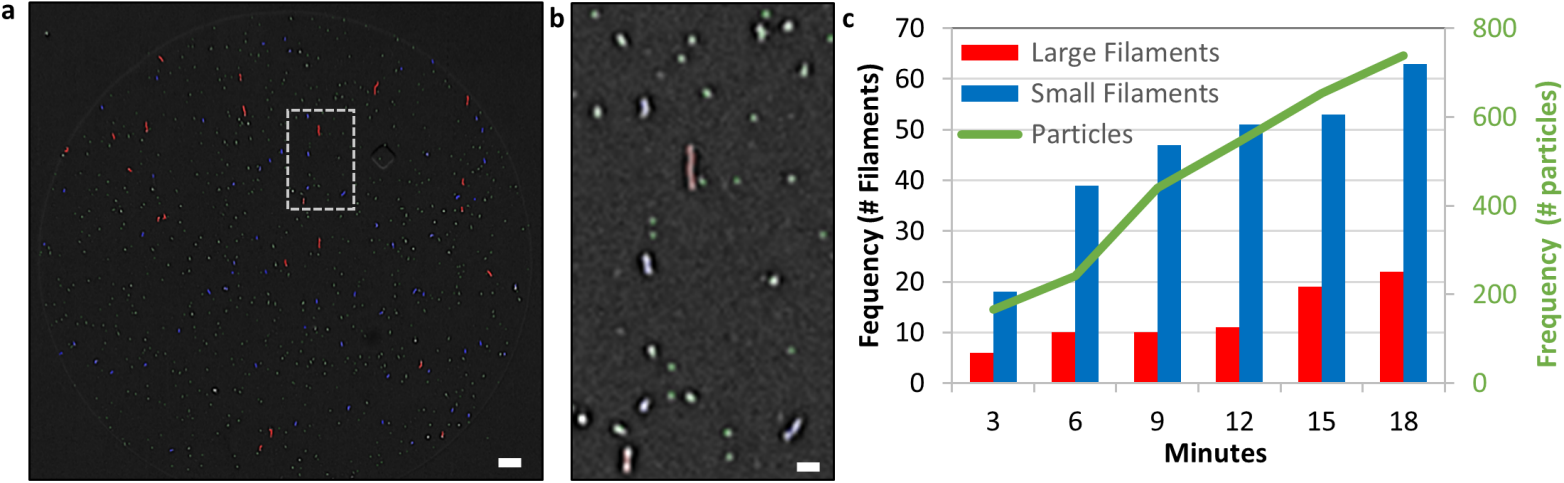
SP-IRIS real-time detection and characterization of Ebola virus in-liquid. a) SP-IRIS image of captured Ebola virus with identified particles and filaments color-coded at eighteen minutes. Scale bar 5 micron b) A zoomed portion of Ebola antibody spot highlighted by square box in 7a. Scale bar 1 micron. c) Plot of the number of small particle and medium (blue) and large (red) filamentous Ebola virions as a function of time.

EBOV particles could also be detected using an in-liquid imaging approach. When serum containing EBOV was placed into a passive-flow IRIS imaging system [14] EBOV (Mayinga isolate) captured by the anti-EBOV GP antibody could be detected in 20 minutes (Fig 8A). Particle size analysis software identified three particle populations: green (nanoparticles), blue (filaments < 1.5 μm), and red (filaments > 1.5 μm). Fig 8B is a zoom-box region of the entire spot (Fig 8A) showing the three different categories of detected particles expressing EBOV GP. Since the SP-IRIS reader is imaging the sensor chip every three minutes the binding can be dynamically monitored. Fig 8C illustrates the binding of the different particle types over time, highlighting similar capture kinetics for all of the particle types.

These results point to a broad applicibility for using the SP-IRIS platform to carry out rapid counting and morphology determination of viruses from different biological fluids based on specific capture using probes against surface proteins. The imaging capability of SP-IRIS allows analysis of a wide dynamic range of viral particles from 40 nm in diameter to several microns in length. This is an important advance for detecting unlabeled virus particles and complements EM and AFM microscopy. EM and AFM have the capacity to offer significantly higher resolution images of virus structure and morphology while counting relatively low numbers of particles. IRIS offers less detailed images of virion structure but offers a much larger field-of-view that allows the enumeration of a much greater number of particles, and functions within a light-microscopy setup that offers additional imaging options. An interesting next step will be to combine SP-IRIS imaging with fluorescence-based labeling. This will allow the probing of multiple aspects of captured virion particles, including presence of genomes within virion particles and the labeling of additional membrane associated proteins.

Another strength of this approach comes from the ease of application. SP-IRIS is an embodiment of light microscopy and so is a low-power approach that can be used on a benchtop in almost any environment. Here we have used it under BSL2 and BSL4 conditions. The approach also has some limitations. It is not able to distinguish viral particles that are positioned within the lateral resolution of the microscope (340-to-435 nm) and so high concentrations of particles can “run together” to create the appearance of larger particles. Therefore, to minimize false sizing and morphology analyses, concentrated viral particle solutions need to be diluted to an acceptable density (< 200,000 particles/mm^2^) when the measurement is performed. This limitation can be overcome via real-time dynamic monitoring of virus binding. With real-time analysis, virus accumulates on the sensor surface allowing tracking and imaging of virus particles as they bind, and tracking of each particle can deconvolute what would otherwise appear to be larger virion particles.

We have presented here the ability of this technology to recognize individual virus particles captured by virus-specific probes. It is exciting to consider the additional approaches for capture and analysis presented by this approach. Using the imaging mode in real-time tracking mode, the approach could be used to follow the fate of individual virus particles bound to antibodies or any other capture ligand. The approach is also likely to be useful in creating histogram based “fingerprints” of virus stocks that can give a direct and ongoing sense of the distribution of virus particles grown in different cells or obtained from clinical samples. This can provide an important additional level of characterization of virion populations that can inform vaccine quality, virus stock quality, and changes in virion populations over time and potentially adaptation to cell culture.

## Supporting information

S1 Fig Detection of 30nm polystyrene particles(PS) by SP-IRIS. a) 30nm diameter PS particles imaged on SP-IRIS. b) SP-IRIS sensor with 30nm PS particle imaged by SEM for verification. Yellow arrows show a sample of particles detected by both system and verifies that individual particles are being detected. c) Zoomed in SEM image shows size verification of particles by SEM.
(TIF)

S2 Fig SP-IRIS image baseline noise. Theoretical shot-noise simulation versus measured noise in SP-IRIS image as a function of number of averaged images. Based on the Rose criterion the minimum signal which can be detected is 5 times the background noise of .1%.
(TIF)

S3 Fig SP-IRIS image of EBOV VLP bound to the anti-EBOV GP antibody on the sensor. The analysis software highlights the detected particles and categorizes them into nanoparticles (green), filaments < 1.5 μm (blue), and long filaments (red). The histogram shows the number of particles detected versus filament length.
(TIF)

S4 Fig SP-IRIS image of EBOV VLP(4cis) bound to the anti-EBOV GP antibody on the sensor. The analysis software highlights the detected particles and categorizes them into nanoparticles (green), filaments < 1.5 μm (blue), and long filaments (red). The histogram shows the number of particles detected versus filament length.
(TIF)

S5 Fig SP-IRIS image of EBOV VLP(4cis-VP24) bound to the anti-EBOV GP antibody on the sensor. The analysis software highlights the detected particles and categorizes them into nanoparticles (green), filaments < 1.5 μm (blue), and long filaments (red). The histogram shows the number of particles detected versus filament length.
(TIF)

S6 Fig SP-IRIS image of EBOV VSV pseudotype bound to the anti-EBOV GP antibody on the sensor. The analysis software highlights the detected particles and categorizes them into nanoparticles (green), filaments < 1.5 μm (blue), and long filaments (red). The histogram shows the number of particles detected versus filament length.
(TIF)

S7 Fig SP-IRIS image of EBOV (Kikwit Strain) bound to the anti-EBOV GP antibody on the sensor. The analysis software highlights the detected particles and categorizes them into nanoparticles (green), filaments < 1.5 μm (blue), and long filaments (red). The histogram shows the number of particles detected versus filament length.
(TIF)
