# Supplementary figures and images for: Enhanced light microscopy visualization of virus particles from Zika virus to filamentous ebolaviruses
Source: PLoS One. 2017 Jun 26;12(6):e0179728. doi: 10.1371/journal.pone.0179728 (PMC5484481; doi:10.1371/journal.pone.0179728)

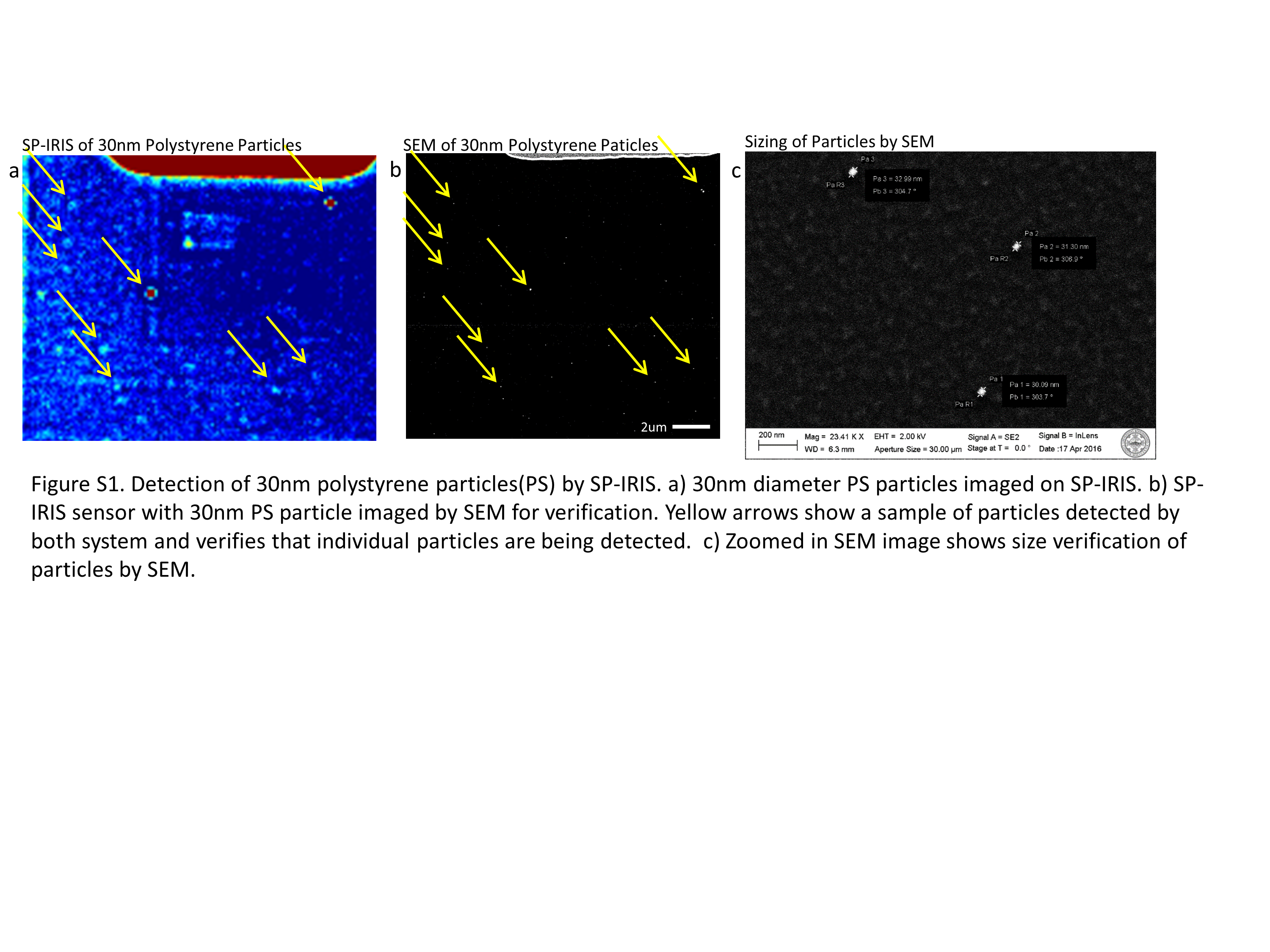

Supplement: S1 Fig — a) 30nm diameter PS particles imaged on SP-IRIS. b) SP-IRIS sensor with 30nm PS particle imaged by SEM for verification. Yellow arrows show a sample of particles detected by both system and verifies that individual particles are being detected. c) Zoomed in SEM image shows size verification of particles by SEM. (TIF) [file pone.0179728.s001.TIF]

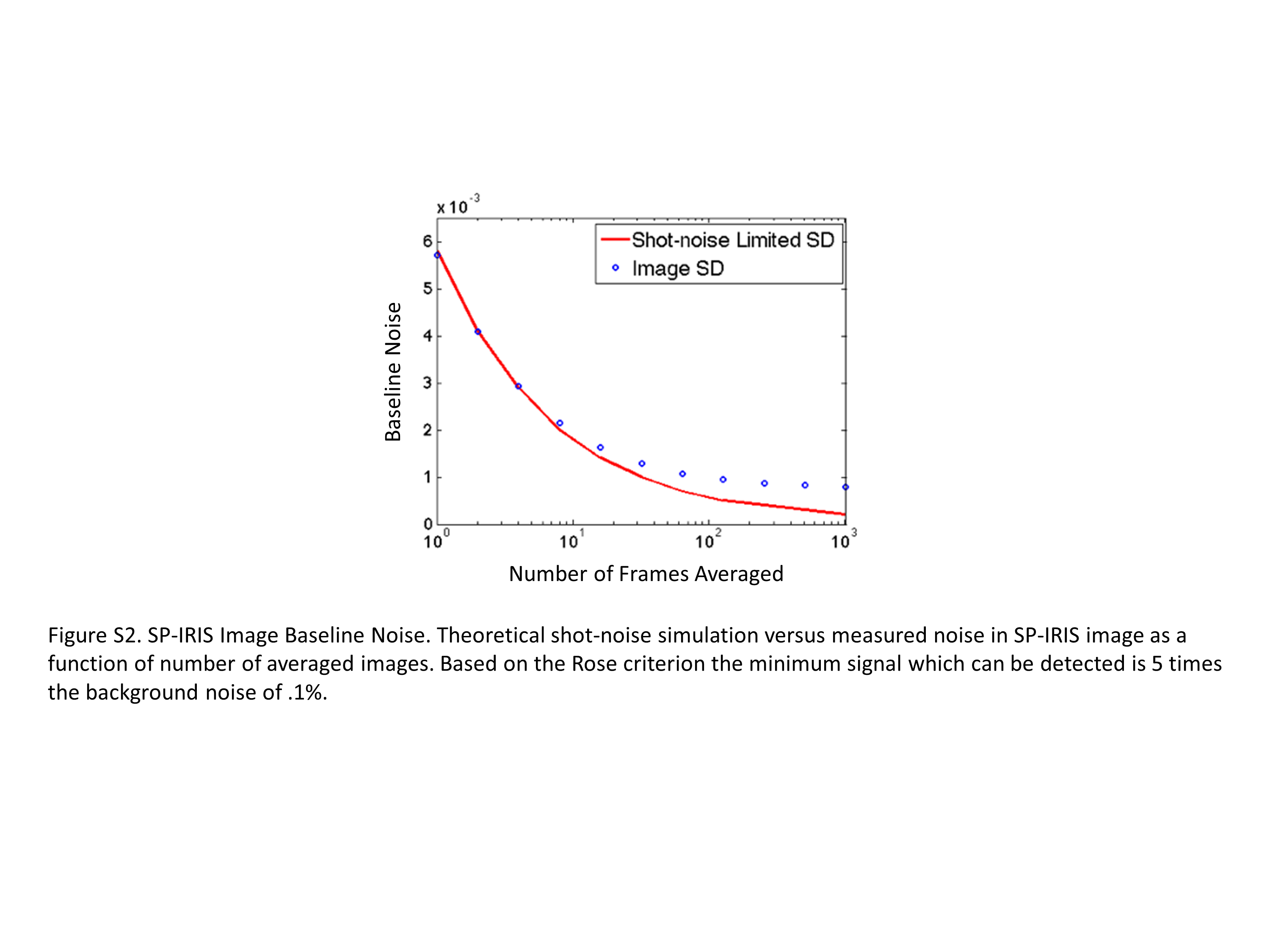

Supplement: S2 Fig — Theoretical shot-noise simulation versus measured noise in SP-IRIS image as a function of number of averaged images. Based on the Rose criterion the minimum signal which can be detected is 5 times the background noise of .1%. (TIF) [file pone.0179728.s002.TIF]

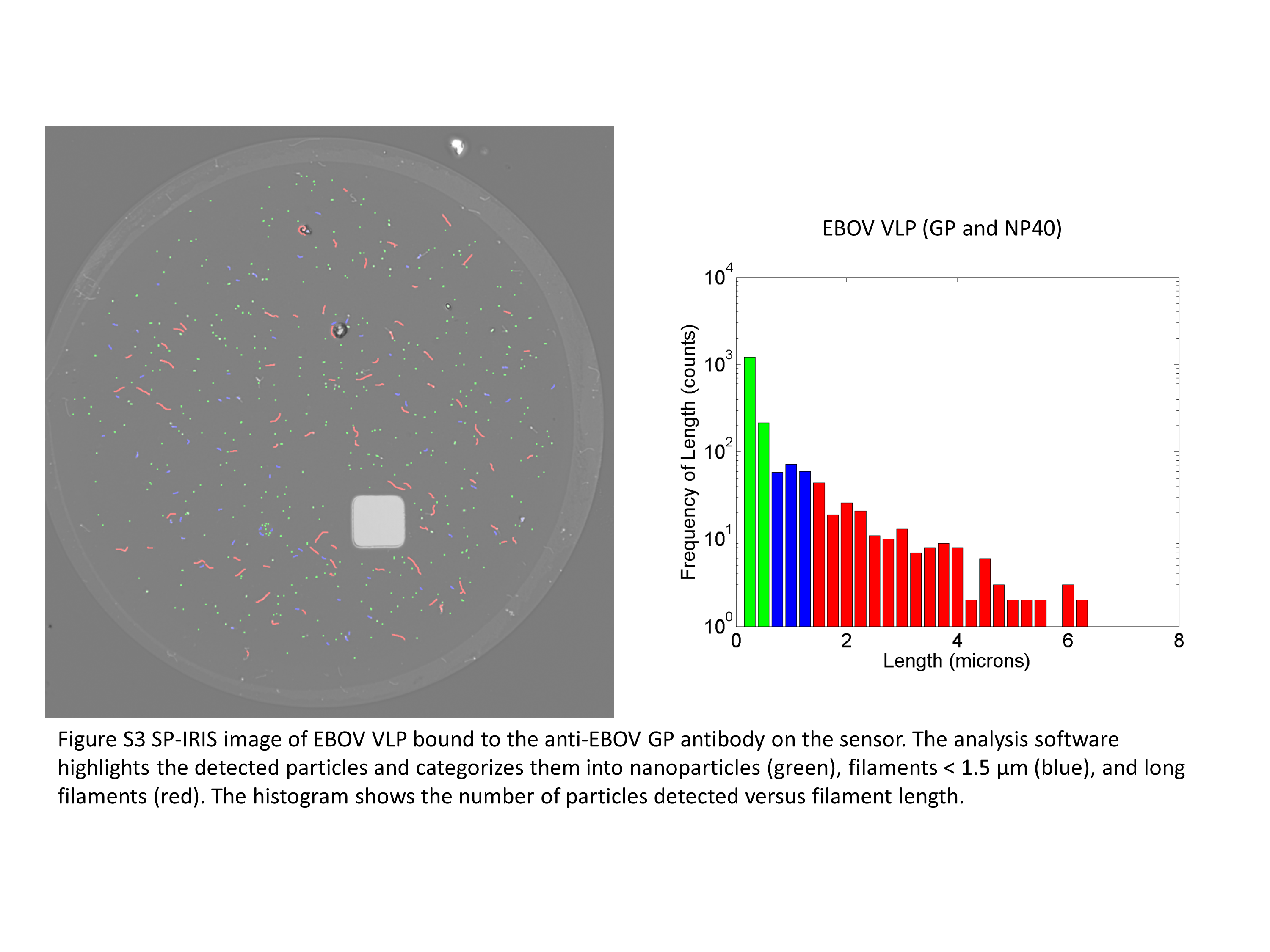

Supplement: S3 Fig — The analysis software highlights the detected particles and categorizes them into nanoparticles (green), filaments < 1.5 μm (blue), and long filaments (red). The histogram shows the number of particles detected versus filament length. (TIF) [file pone.0179728.s003.TIF]

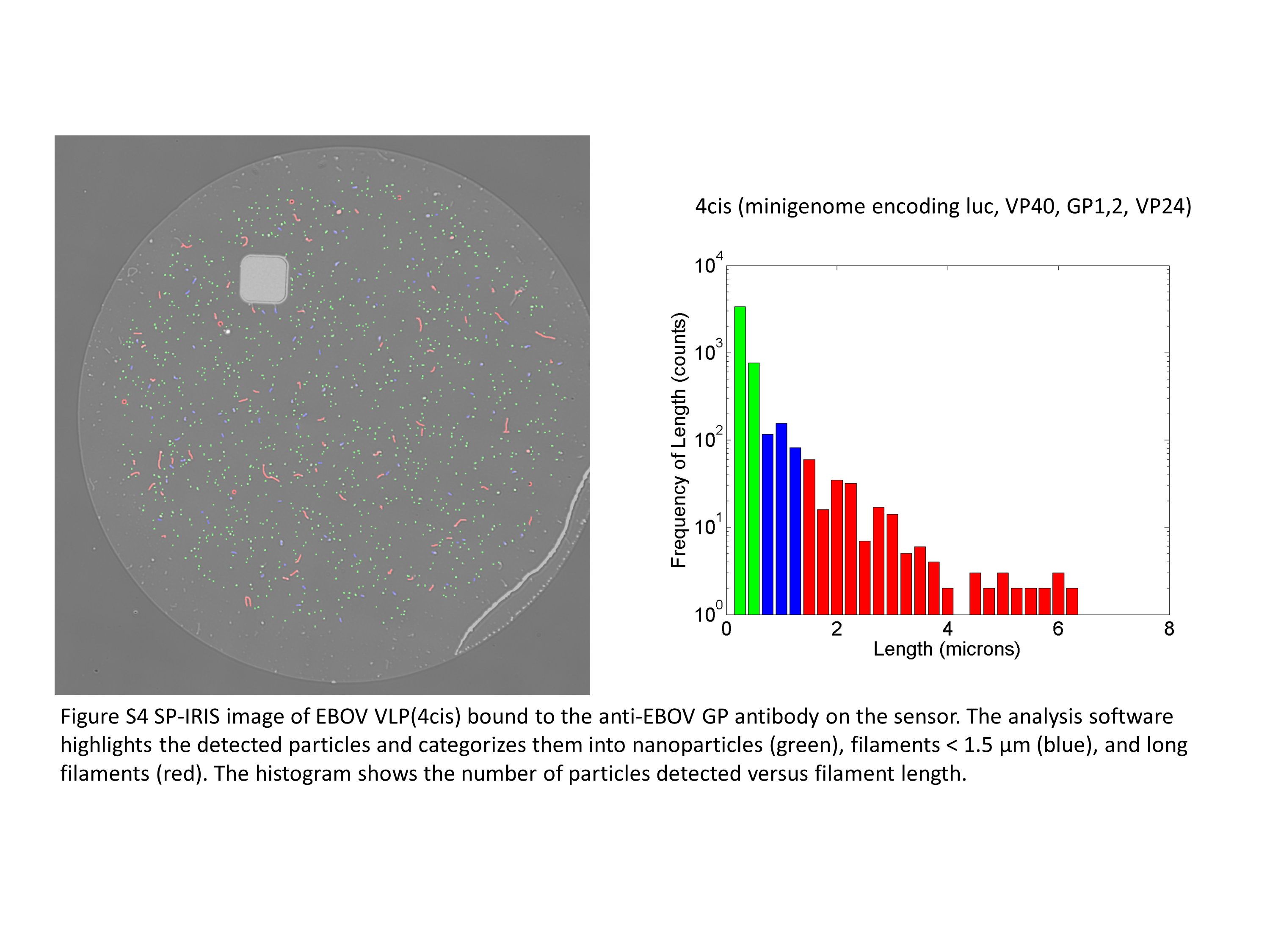

Supplement: S4 Fig — The analysis software highlights the detected particles and categorizes them into nanoparticles (green), filaments < 1.5 μm (blue), and long filaments (red). The histogram shows the number of particles detected versus filament length. (TIF) [file pone.0179728.s004.TIF]

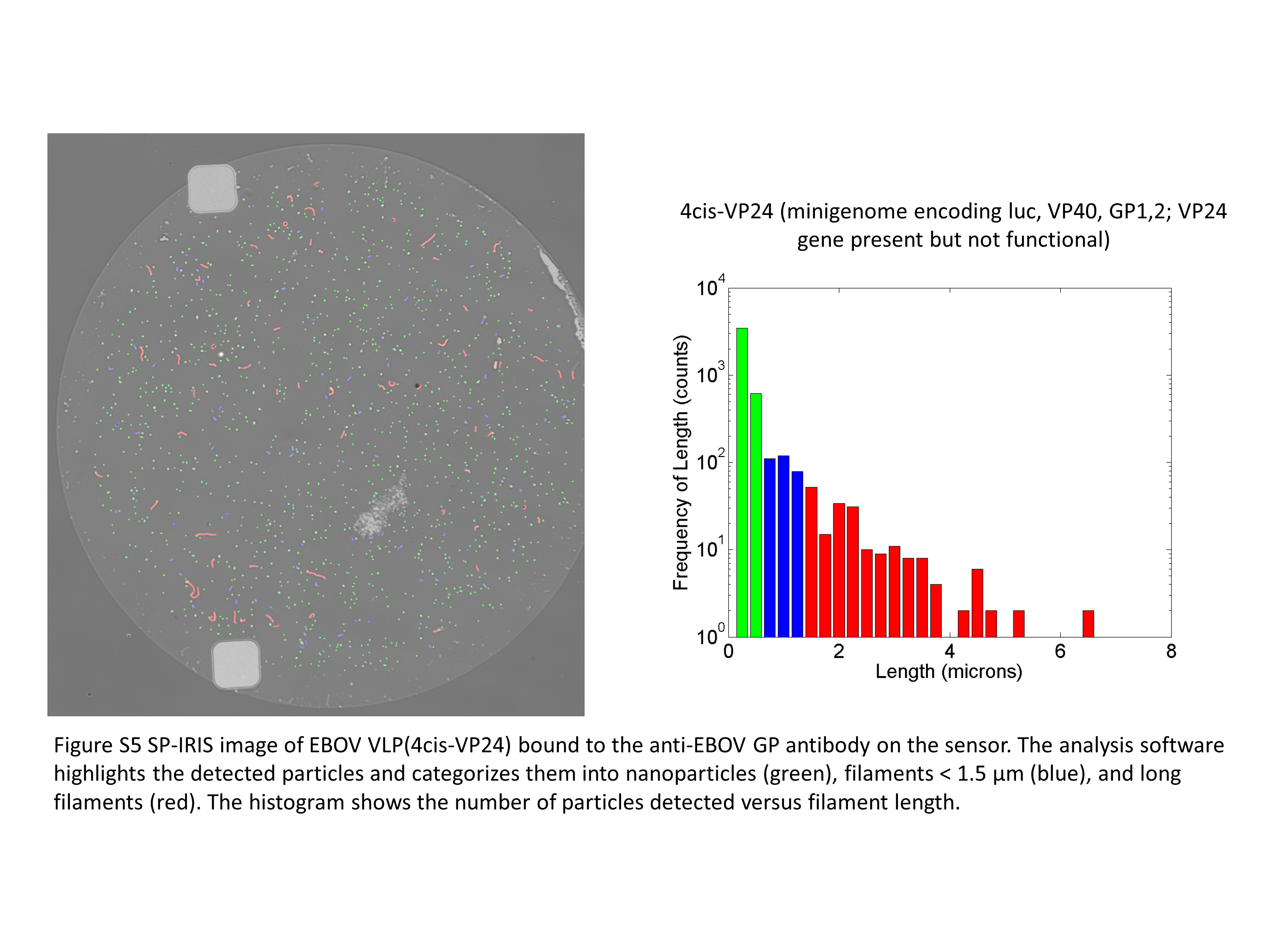

Supplement: S5 Fig — The analysis software highlights the detected particles and categorizes them into nanoparticles (green), filaments < 1.5 μm (blue), and long filaments (red). The histogram shows the number of particles detected versus filament length. (TIF) [file pone.0179728.s005.TIF]

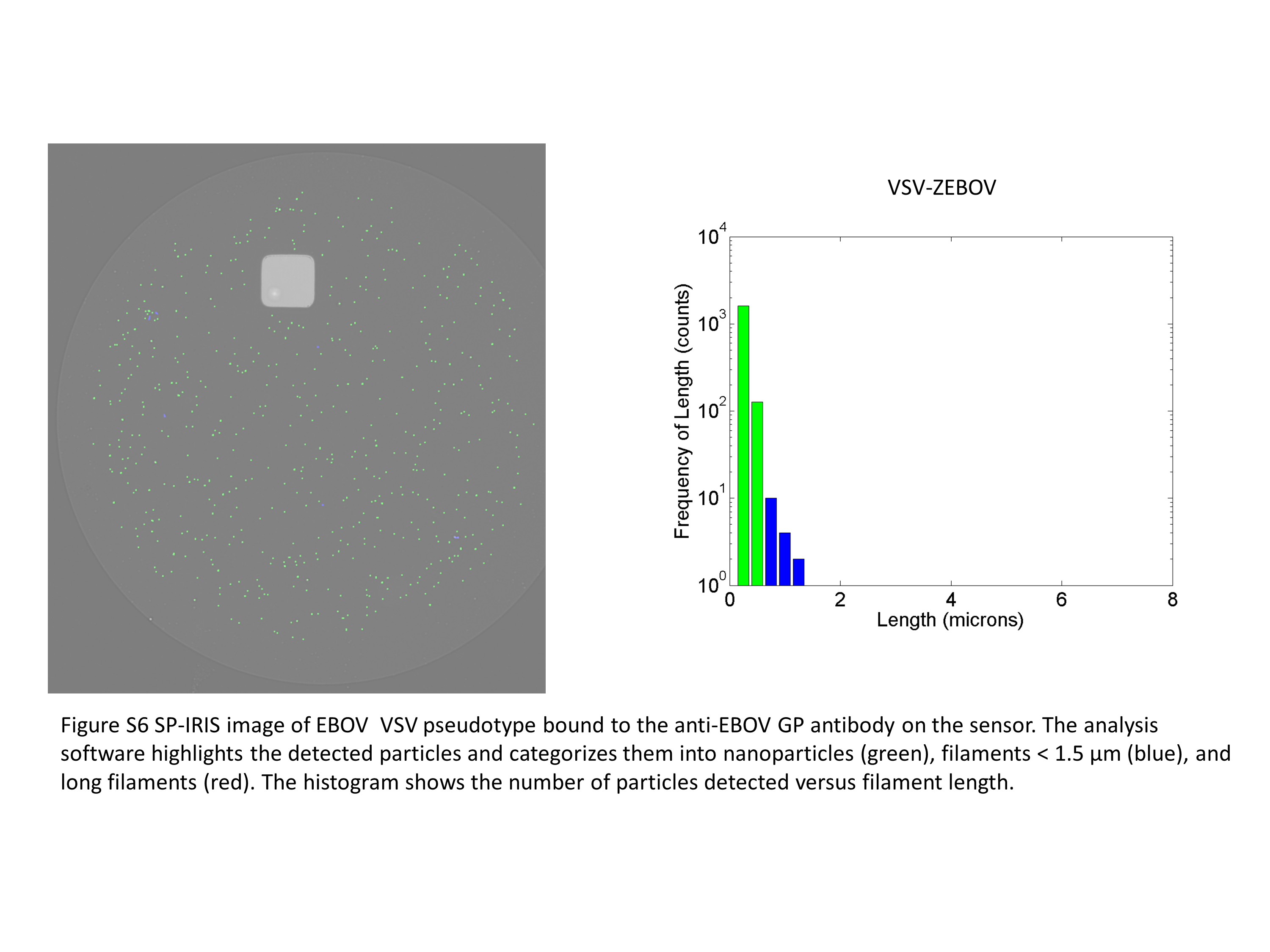

Supplement: S6 Fig — The analysis software highlights the detected particles and categorizes them into nanoparticles (green), filaments < 1.5 μm (blue), and long filaments (red). The histogram shows the number of particles detected versus filament length. (TIF) [file pone.0179728.s006.TIF]

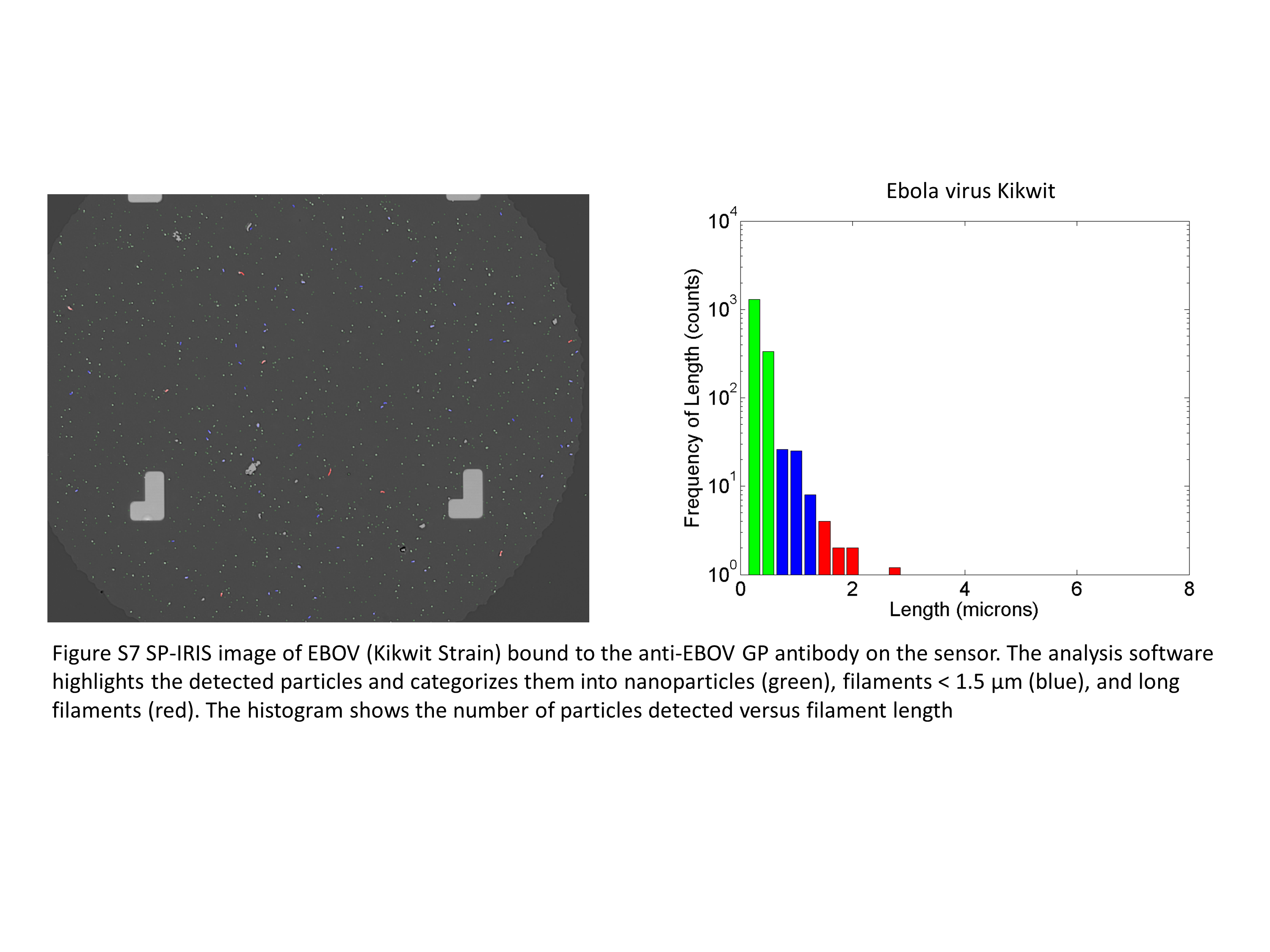

Supplement: S7 Fig — The analysis software highlights the detected particles and categorizes them into nanoparticles (green), filaments < 1.5 μm (blue), and long filaments (red). The histogram shows the number of particles detected versus filament length. (TIF) [file pone.0179728.s007.TIF]
